# Life history traits in selfing versus outcrossing annuals: exploring the 'time-limitation' hypothesis for the fitness benefit of self-pollination

**DOI:** 10.1186/1472-6785-5-2

**Published:** 2005-02-11

**Authors:** Rebecca Snell, Lonnie W Aarssen

**Affiliations:** 1 Dept of Biology, Queen's Univ., Kingston, ON, K7L 3N6, Canada

## Abstract

**Background:**

Most self-pollinating plants are annuals. According to the 'time-limitation' hypothesis, this association between selfing and the annual life cycle has evolved as a consequence of strong r-selection, involving severe time-limitation for completing the life cycle. Under this model, selection from frequent density-independent mortality in ephemeral habitats minimizes time to flower maturation, with selfing as a trade-off, and / or selection minimizes the time between flower maturation and ovule fertilization, in which case selfing has a direct fitness benefit. Predictions arising from this hypothesis were evaluated using phylogenetically-independent contrasts of several life history traits in predominantly selfing versus outcrossing annuals from a data base of 118 species distributed across 14 families. Data for life history traits specifically related to maturation and pollination times were obtained by monitoring the start and completion of different stages of reproductive development in a greenhouse study of selfing and outcrossing annuals from an unbiased sample of 25 species involving five pair-wise family comparisons and four pair-wise genus comparisons.

**Results:**

Selfing annuals in general had significantly shorter plant heights, smaller flowers, shorter bud development times, shorter flower longevity and smaller seed sizes compared with their outcrossing annual relatives. Age at first flower did not differ significantly between selfing and outcrossing annuals.

**Conclusions:**

This is the first multi-species study to report these general life-history differences between selfers and outcrossers among annuals exclusively. The results are all explained more parsimoniously by selection associated with time-limitation than by selection associated with pollinator/mate limitation. The shorter bud development time reported here for selfing annuals is predicted explicitly by the time-limitation hypothesis for the fitness benefit of selfing (and not by the alternative 'reproductive assurance' hypothesis associated with pollinator/mate limitation). Support for the time-limitation hypothesis is also evident from published surveys: whereas selfers and outcrossers are about equally represented among annual species as a whole, selfers occur in much higher frequencies among the annual species found in two of the most severely time-limited habitats where flowering plants grow – deserts and cultivated habitats.

## Background

Most flowering plants that are predominantly self-pollinating have an annual life history [1-3]. Interpretations of this association usually involve one of two main hypotheses. (*i*) Compared with perennials, annuals may generally accrue greater fitness benefits from selfing through 'reproductive assurance', i.e., because ovules may be generally more outcross-pollen-limited and/or pollen grains may be more outcross-ovule-limited [2,4-8]. (*ii*) Perennials may incur a higher fitness cost of selfing through seed discounting and inbreeding depression; hence, possibly most selfers are annuals simply because relatively few perennials can be selfers [9,10].

A recent third hypothesis, the 'time-limitation' hypothesis, predicts that both selfing and the annual life cycle are concurrent products of strong 'r-selection' associated with high density-independent mortality risk in ephemeral habitats with a severely limited period of time available to complete the life cycle [11]. Both the traditional reproductive assurance hypothesis and the time-limitation hypothesis involve a fitness advantage for selfing through ensuring that at least some reproduction occurs, but they involve very different selection mechanisms – *pollinator/mate-limitation *(where outcross pollen is not available at all due to a lack of pollinators or mates), versus *time-limitation *(where outcross pollen is available but arrives too late to allow sufficient time for development of viable seeds). Accordingly, these two hypotheses for selfing involve very different assumptions and predictions.

**Figure 1:**
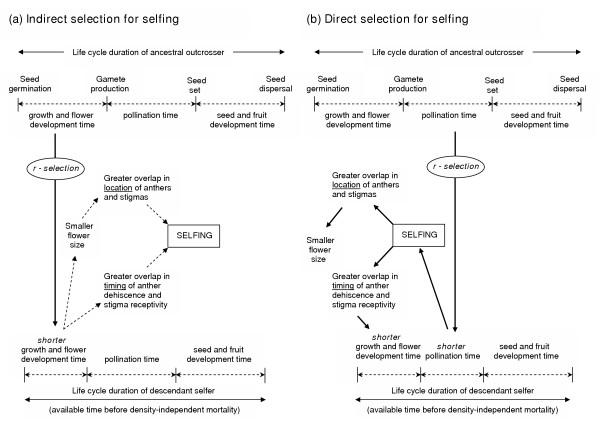
Two components of the 'time-limitation' hypothesis for the evolution selfing in annuals. In (a), selfing is a trade-off of selection favoring a shorter time to reproductive maturity (fully developed flowers) under strong r-selection. As a tradeoff (dashed arrows), flowers become smaller with greater overlap in location and timing of anther dehiscence and stigma receptivity, thus increasing the rate of selfing as an incidental consequence. In (b) strong r-selection favors a shorter pollination time directly; i.e., selfing is selected for directly because it shortens the amount of time between flower maturation and ovule fertilization, thus leaving sufficient remaining time for seed and fruit maturation before the inevitable early mortality of the maternal plant under strong r-selection. In this case, smaller flower size and shorter flower development time are favored by selection because they facilitate selfing (see text).

The time-limitation hypothesis has direct and indirect components. The indirect component predicts higher selfing rates in annuals as a trade-off of selection for earlier reproductive maturity in annuals [12,13] (Figure 1a). More rapid floral maturation is expected to result in smaller flowers with increased overlap of anther dehiscence and stigma receptivity in both space (reduced herkogamy) and time (reduced dichogamy) thus, increasing the frequency of selfing as an incidental consequence [12] (Figure 1a). If selfing also shortens the time between flower maturation and ovule fertilization, then higher selfing rates for annuals in time-limited habitats may also be predicted as a *direct *fitness benefit; abbreviating the time between anthesis and ovule fertilization may ensure that there is enough remaining time in the growing season (after ovule fertilization) to allow complete seed and fruit maturation [11] (Fig 1b). Selection favors selfing here by favoring increased overlap in anther dehiscence and stigma receptivity in both space and time, which are in turn facilitated by smaller flower size and shorter flower development time, respectively (Figure 1b).

However, the two components of time-limitation cannot be separated clearly, as they operate simultaneously; i.e., earlier onset of flowering, shorter flower development time, smaller flowers and selfing can all be interpreted to have direct fitness benefits because they may all contribute directly to accelerating the life cycle [11]. Indeed, time-limitation associated with strong r-selection would be expected also to favor an acceleration of the final stage in the life cycle – seed/fruit development time (Figure 1) – resulting, as a trade-off, in smaller seeds and/or fruits [11].

The time-limitation hypothesis remains untested. Some recent studies have explored the rapid growth and maturation time of annuals in terms of bud development rates and ontogeny [13-15]. However, these studies have compared growth and development rates between selfing and outcrossing populations of only a single species. Since their effective sample size is only one, this makes it difficult to extrapolate the predominant selection pressures that may have promoted the general association of selfing with the annual life cycle.

The objective of the present study was to compare, for annuals exclusively, life history traits associated with selfing versus outcrossing using several species from a wide range of plant families. Phylogenetically-independent contrasts (PIC) were used to control for confounding effects due to common ancestry among species [16]. Using a database of 118 species involving 14 families, plant size, flower size, and seed size were compared between selfing and outcrossing annuals. The time-limitation hypothesis predicts that all of these traits should be smaller in selfing annuals because the severely time-limited growing season that promotes selfing also imposes an upper limit on the maximum sizes that can be attained for plant traits [11] (Figure 1). The trend for outcrossers to be taller, and have larger flowers and larger seeds has often been noted [1,17-19]. We used a multi-species, across-family comparison, however, to investigate whether this trend also holds true within annuals exclusively.

Data on the timing of life history stages (i.e. age at first flower, bud development time, and flower longevity) were also obtained from a greenhouse study of 25 annual species involving 5 families. The time-limitation hypothesis predicts that selfers should produce mature flowers more quickly and should have shorter flowering times.

## Results

### Data base analyses

**Figure 2:**
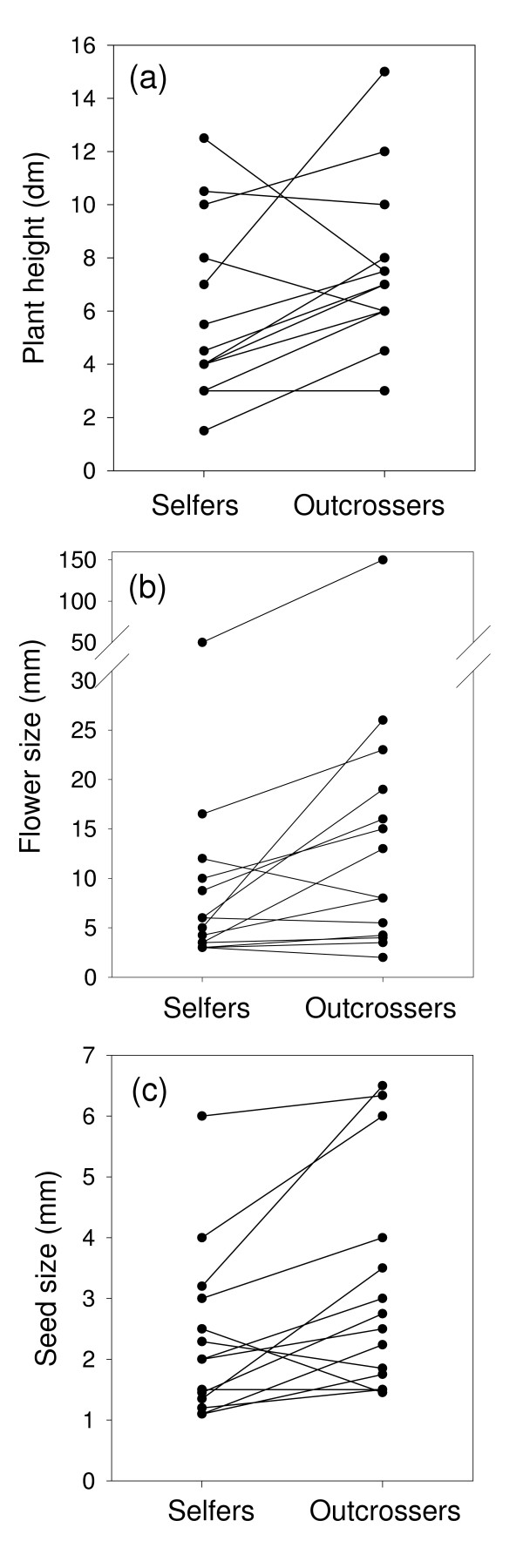
(a) Plant height contrasts for 13 selfing and outcrossing pairs (some points overlap), where each pair consists of the median value of the selfing and outcrossing species within one family. (b) Flower size contrasts for 14 selfing and outcrossing pairs. (c) Seed size constrasts between 14 selfing and outcrossing pairs. (See Appendix A for list of families and species).

Based on phylogenetically-independent contrasts, selfing annuals had significantly shorter plant heights (Wilcoxon test for matched pairs, n = 12, T = 15.5, one-tailed P = 0.032, Figure 2a), significantly smaller flowers (Wilcoxon test for matched pairs, n = 14, T = 13, one-tailed P = 0.0054, Figure 2b), and significantly smaller seeds (Wilcoxon test for matched pairs, n = 13, T = 13, one-tailed P < 0.01, Figure 2c).

### Greenhouse study

**Figure 3:**
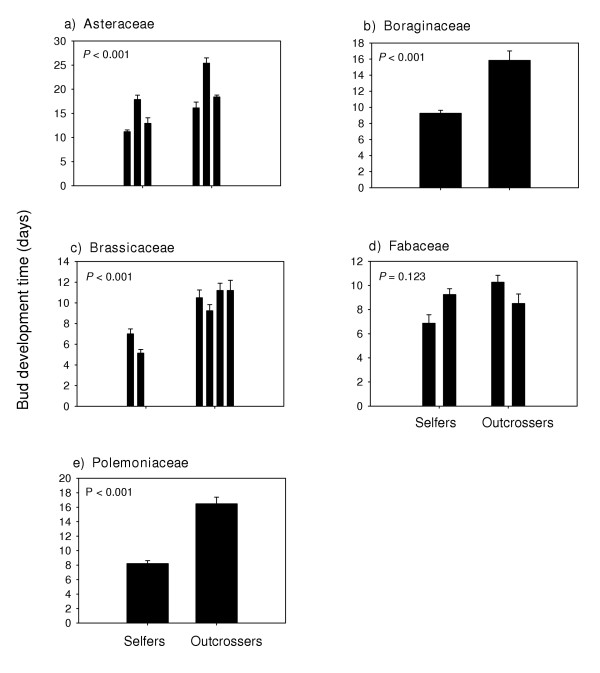
Mean (SE) bud development time for selfing and outcrossing species within each of 5 families. For each species, n = 4 or 5. *P *– values are from ANOVA. (See Appendix B for species list).

**Figure 4:**
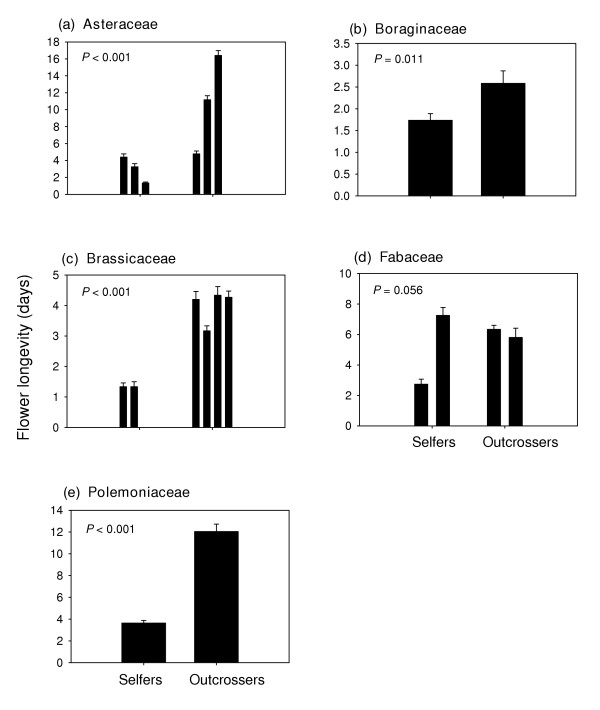
Mean (SE) flower longevity for selfing and outcrossing species within each of 5 families. For each species, n = 4 or 5. *P *– values are from ANOVA. (See Appendix B for species list).

**Figure 5:**
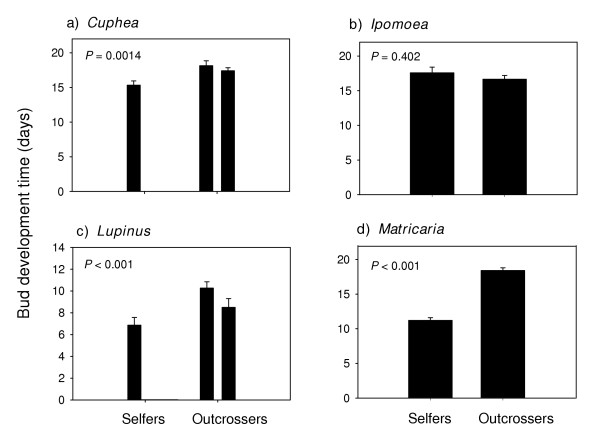
Mean (SE) bud development time for selfing and outcrossing species within each of 4 genus pairs. For each species, n = 4 or 5. *P *– values are from ANOVA. (See Appendix B for species list).

**Figure 6:**
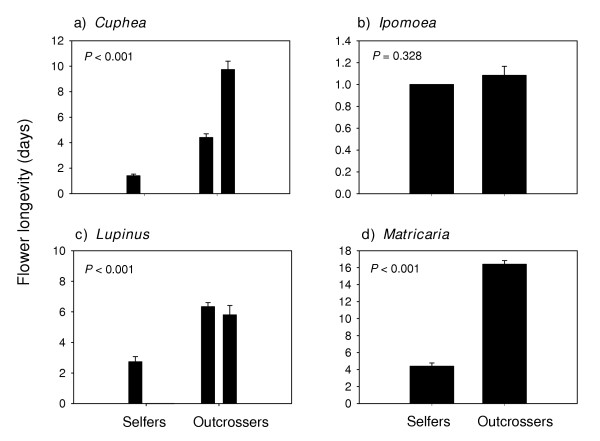
Mean (SE) flower longevity for selfing and outcrossing species within each of 4 genus pairs. For each species, n = 4 or 5. P – values are from ANOVA. (See Appendix B for species list).

Bud development time (Figure 3) and flower longevity (Figure 4) were significantly (P < 0.05) shorter in selfing annuals in all of the families except the Fabaceae (P = 0.123 and P = 0.056 respectively). Selfing annuals also had significantly shorter bud development times (Figure 5), and floral longevities (Figure 6) in three of the four genus pairs. Selfing and outcrossing annuals of the genus *Ipomoea *did not differ significantly in either bud development time (P = 0.402) or flower longevity (P = 0.328). Age at first flower was not significantly related to mating system for any of the family or genus comparisons (P > 0.05; data not shown).

## Discussion

There is a rich body of theory and empirical work on the evolution of selfing in flowering plants [e.g. [1,2,4-7,9,10]], but practically none of it involves an explicit role of selection involving time-limitation. The present paper is only the second to explore the implications of the time-limitation hypothesis and contribute to the maturation of this idea. According to the time-limitation hypothesis, selfing in annuals has evolved as a consequence of strong r-selection in ephemeral habitats, resulting either as an indirect consequence (trade-off) of selection for shorter time to reproductive maturity (Figure 1a), or as a direct consequence of selection for shorter pollination time, i.e., the time between flower maturation and ovule fertilization (Figure 1b), or both [11]. Consistent with the predictions of this hypothesis, we found, using phylogenetically-independent contrasts, that (compared with outcrossing annuals) selfing annuals in general had significantly shorter plant heights, smaller flowers, shorter bud development time, shorter flower longevity and smaller seed sizes.

At the same time, these results are not inconsistent with the predictions of selection resulting from pollinator/mate-limitation associated with the traditional reproductive assurance hypothesis. Just as with many situations where two different mechanisms can potentially produce the same outcome/pattern, it is not easy here to clearly distinguish between the roles of "pollinator/mate-limitation" and "time-limitation". Nevertheless there are two important contributions from our study: First, in reporting significant life history differences between selfers and outcrossers, our multi-species study is unique in its comparison of monocarpic annual species exclusively. All previous multi-species studies of trait comparisons between selfers and outcrossers have involved variable mixes of monocarpic and longer-lived polycarpic species. Second, by comparing annuals exclusively, our results provide indirect support for the time-limitation hypothesis, not by rejecting the role of pollinator/mate-limitation, but rather by representing a system in which it is more plausible to argue for the role of time-limitation; i.e., compared with pollinator/mate-limitation, time-limitation as a selection factor favoring selfing is likely to have been much stronger, more persistent and more widespread. The strength of this argument lies in the fact that the annual life history is unequivocally a product of some type of time-limitation favoring an abbreviated life cycle, which is promoted by (among other things) selfing (as opposed to outcrossing) (Fig. 1). It is much less plausible to suspect that selection associated with pollinator/mate-limitation has been sufficiently strong and persistent to favor selfing in such a wide range of annual taxa across the many genera and families considered here. We emphasize, therefore, that for annuals the time-limitation hypothesis provides a more parsimonious explanation for the differences in traits between selfers and outcrossers. We consider each of these traits in turn below.

### Plant height and time to anthesis

Taller plants may attract more pollinators and, hence, experience greater outcrossing rates [20,21]. The pollination benefit of being relatively tall, therefore, is presumably experienced only by outcrossers. If, however, selfers have evolved from outcrossers [3], then why should selfers be shorter than their outcrossing ancestors? The relatively small size, including short height of selfers can be predicted as an indirect consequence of selection, from time-limitation, favoring precocious maturation time [22,23] (Figure 1a). In the present study, however, selfers and outcrossers did not differ significantly in age at first flower. Andersson [18] found similar results between selfing and outcrossing populations of *Crepis tectorum*. Arroyo [24], however, reported that selfing individuals of *Limnanthes floccosa *flowered earlier than the outcrossing *L. alba*, as predicted by the time-limitation hypothesis. The results for flowering times in the present study may be confounded by the controlled greenhouse environment of constant day-length, temperature and moisture regime. In the field, flowering times may be triggered by environmental cues. *L. floccosa*, for example, uses soil moisture to trigger the early onset of flowering, thus escaping the detrimental effects of soil desiccation during seed development [24]. Note also that age at first flower is only a crude estimate of time to reproductive maturity. Future studies may employ more detailed measures such as rate of mature flower production.

### Flower size

One of the most well established trends of predominantly self-fertilizing species is their reduced flower sizes compared with outcrossing species [1,17]. The present results indicate that this trend is also evident even within annuals exclusively. In all but three of the 14 PICs, selfing annuals had smaller flowers than the outcrossing annuals (Figure 2b). Outcrossers and selfers had similar flower sizes in the Fabaceae and Plantaginaceae. In the Poaceae, outcrossing annuals had smaller flowers than selfers.

Under the time-limitation hypothesis, smaller flowers and selfing may be tradeoffs of selection for precocity (Figure 1a), or smaller flowers may be favored by selection because they promote selfing and hence, direct fitness benefits by abbreviating pollination time (Figure 1b). Also, if selfing evolves from outcrossing (by whatever mechanism), then selection may subsequently favour a reduction in flower size since relatively large flowers are no longer needed to attract pollinators. Hence, higher fitness may result if the resources required to construct and support these larger flowers are invested instead in other functions (e.g. seed and fruit development) [17].

### Bud development time

Selfers had significantly shorter bud development times in all but one of the independent family contrasts (Figure 3) and all but one of the genus comparisons (Figure 5). Results from previous studies, however, are inconsistent. Shorter bud development times were found in selfing populations of *Mimulus guttatus *[25] and in *Clarkia xantiana *[14]. However, no significant differences in bud growth rates were found between the selfing and outcrossing populations of *C. tembloriensis *[15]. Hill, Lord and Shaw [13] reported that flowers from selfing populations of *Arenaria uniflora *develop over a longer period of time than observed in outcrossing populations. In the field, selfing populations of *A. uniflora *were also observed to flower at the same time or even later than outcrossing populations [13], suggesting that time-limitation is not currently a strong selection pressure. Self-fertilization in *A. uniflora *may have arisen through reproductive assurance in response to competition for pollinators [7]. The evolution of self-fertilizing species from outcrossing progenitors has occurred repeatedly and independently in several lineages [1,3,14], each of which may have been associated with different contexts of natural selection *vis-à-vis *the fitness benefits of selfing.

### Flower longevity

The families and genera in which selfers had shorter bud development times also had significantly shorter flower longevities (Figure 4). In fact, all of the selfers had flowers that remained open for less than four days (except in *Trifolium hirtum*; Fabaceae), with a large proportion of flowers open for only one day, which is common amongst self-fertilizing species [17]. The present data again indicate that this generalization apparently holds true even within annuals exclusively. By having flowers that remain open longer, outcrossers increase the probability of visitation by pollinators and successful cross-pollination [17]. This fitness benefit is realized, however, only if there is sufficient time remaining after cross-pollination to complete seed and fruit development before the maternal plant succumbs to density-independent mortality in strongly r-selecting habitats [11]. If time is limiting in this context, selection should favor selfing (Figure 1b) with no advantage in having long-lived flowers.

It is important to note that our data measure *maximum *flower longevity, since there were no pollinators in the greenhouse, nor was hand pollination conducted. Pollination has been shown to induce floral senescence in numerous species [26]. This effect was not tested on any of the study species, which means that our observed flower longevities in outcrossing species may be longer than would normally be seen in the wild. Nevertheless, since selfing may have evolved as a method of shortening pollination time, and flower longevity was used as a measure of pollination time, the maximum floral longevity gives an indication of how long outcrossers can delay flower abscission or self-pollination (i.e. through delayed selfing).

### Seed size

Strong r-selection associated with the annual life form presumably favors wide dispersal mechanisms (for colonizing new and distant sites) which may be conferred by small seed sizes [19]. The reproductive assurance hypothesis would predict, therefore, that most selfers are annuals because annuals are more likely than perennials to disperse further, or colonize new habitats where conditions are unsuitable for successful outcrossing (because of a shortage of mates or pollinators) and where selfing, therefore, provides reproductive assurance. The present study indicates that even among annuals only, selfers have smaller seeds than outcrossers (Figure 2c). Future studies are required to test whether smaller-seeded selfing annuals are more likely than their outcrossing annual relatives to disperse further or colonize new habitats and thereby incur potential reproductive assurance benefits of selfing.

An alternative explanation, however, is offered by an extension of the time-limitation hypothesis: strong r-selection favors an acceleration of all stages of the life cycle (Figure 1), including not only earlier reproductive maturity (Figure 1a) and a shorter pollination time (facilitated through selfing) (Figure 1b), but also a shorter seed and fruit maturation time, which, on a per-seed basis, is facilitated in turn through the production of smaller seeds. Andersson [18] found that self-fertilizing individuals of *Crepis tectorum *took an average of 16 days for fruit maturation, whereas outcrossing individuals of the same species required 43.3 days. Small seed size may also be simply a trade-off of selection for high fecundity, also favored by strong r-selection [11].

### Habitat selection and time-limitation

While most selfers are annuals, it is not the case that most annuals are selfers. An unbiased literature survey [27] suggests that roughly half of all annual species are selfers and half are outcrossers. If, however, selfing annuals evolved in habitats with a short window of time for completing the life cycle (Figure 1), then selfing annuals should be significantly more common than expected (i.e. comprising greater than 50% of resident annuals) within habitats associated with historically regular, early-season disturbances (e.g. cultivated fields, gardens), or in habitats where severe droughts follow quickly after a wet season (i.e. deserts, Mediterranean climates, vernal pools). Hence, we should expect to find more selfers than outcrossers among annual weeds of cultivated habitats and among desert annuals in particular. Similarly, for annuals with both selfing and outcrossing ecotypes or races, we should expect selfers (or a higher selfing rate) to be more commonly associated with these severely time-limited habitats [11].

While rigorous tests of these predictions have yet to be explored, some preliminary support is available from published surveys. From a representative sample of Mediterranean annuals [28], we find a much greater representation of selfers: i.e. 34 selfers versus 11 outcrossers. Selfing and outcrossing desert annuals have been shown to be distributed along a moisture gradient. Outcrossing annuals are found generally in the wetter areas and selfers in the more arid zones, as seen in *Clarkia xantiana *[29] and between outcrossing populations of *Limnanthes alba *and its selfing relative *L. floccosa *[24]. Since the length of the growing season is limited by the amount of moisture in the soil, selfers have a much narrower window of time to complete their life cycle before desiccation. During a severe drought, seed production in *L. alba *was reduced by one sixth, whereas the seed set of *L. floccosa *found in the same area was virtually unaffected by the identical drought [24].

The association between 'weediness' and self-fertilization has also been noted [2,30]. An extensive survey of colonizing herbaceous plants of Canada showed that agricultural weeds of row crops and grain fields are almost exclusively annuals, and most of these are self-compatible [31]. A published list of the world's worst weeds of agricultural crops [32] includes 76 species, 41 of which are annuals. Based on previous literature, we were able to identify the breeding system for 24 of these annuals, and, as predicted, the majority (20 out of 24) are selfers.

## Conclusions

Botanists have long known that selfing is particularly associated with the annual life cycle in flowering plants [2]. The present study shows further that, among annuals exclusively, selfing is particularly associated with shorter plant heights, smaller flowers, shorter bud development time, shorter flower longevity and smaller seed sizes compared with annuals that are outcrossing. Also, in spite of the null prediction that selfing and outcrossing annuals should be equally represented if there is no bias associated with time-limitation, we found instead that two of the most time-limited habitats on earth that support flowering plants have a significantly higher percentage of selfers among the resident species that are annuals. Because we focused on annual species only, all of these results are explained more parsimoniously by selection associated with time-limitation than by selection associated with pollinator/mate limitation. The role of pollinator/mate-limitation (as traditionally associated with the reproductive assurance hypothesis for the evolution of selfing) is likely to be of greater importance in longer-lived polycarpic species (not considered here), simply because by comparison, there is no convincing basis to argue that selection associated with time-limitation is likely to have been important in species with longer life cycles. We suggest therefore, that most selfers, because most of them are annuals, are likely to have evolved not because of fitness benefits through reproductive assurance associated with selection from pollinator/mate limitation, but rather because of fitness benefits associated with selection from time limitation.

The effect of time-limitation under strong r-selection is to minimize the duration of the life cycle, with selfing favored directly (Figure 1b) and/or indirectly (Figure 1a). There is no basis for predicting that either mechanism is more probable than the other; both are likely to operate simultaneously and perhaps indistinguishably. Indeed, the predicted effects under direct and indirect selection involve the same phenotypic outcome for the same suite of traits (Figure 1). It is particularly significant that the shorter bud development time reported here for selfing annuals is predicted explicitly by the time-limitation hypothesis but not by selection associated with pollinator/mate limitation. Although, we cannot of course rule out the possibility that shorter bud development time may be a pleiotropic consequence of the evolution of autonomous selfing through other mechanisms.

Designing empirical studies that clearly distinguish between mechanisms involving time-limitation versus pollinator/mate limitation remain a challenge but we anticipate that our results and our discussion of these issues may help to inspire further research along these lines. Future studies may be designed to test more directly the role of limited pollination time (*vis-à-vis *Figure 1b) by comparing the time required for effective pollination under selfing versus outcrossing for closely related species or ecotypes within natural habitats, taking care of course to control for other aspects of the pollination environment (such as mate and pollinator availability) that might affect time-to-effective pollination.

## Methods

### Data base analyses

The literature was surveyed to obtain breeding information (i.e. selfing versus outcrossing) for as many annuals species as possible. For each species, data on plant height, flower size, and seed size were obtained where possible from standard floras and other published literature. A complete database was assembled for 118 species from both Europe and North America, involving 14 families (Table 1). For each species, the maximum published value for each trait was used. Plant height was the maximum recorded vertical extent of the plant. The measure used for flower size depended on the usual convention specific for each family, e.g. maximum petal length, corolla width, lemma length (in the Poaceae). Seed size was measured as the length of the longest axis. Within each family, for each trait, the median value across selfing species and the median value across outcrossing species was calculated and used in the phylogenetically-independent contrasts.

**Table 1:** Species list, with breeding system (O – outcrosser; S – selfer), for database from published literature.

Family	Family
Species	Species
Asteraceae	Malvaceae
*Anthemis cotula *(O)	*Abutilon theophrasti *(S)
*Cosmos bipinnatus *(O)	*Hibiscus trionum *(O)
*Centaurea cyanus *(O)	*Malva neglecta *(O)
*Centaurea montana *(O)	*Malva rotundiflora *(S)
*Crepis capillaris *(O)	
*Crepis tectorum *(O)	Plantaginaceae
*Helianthus annuus *(O)	*Plantago arenaria *(O)
*Lapsana communis *(O)	*Plantago virginica *(S)
*Matricaria maritima *(O)	
*Matricaria matricarioides *(S)	Poaceae
*Senecio viscosus *(S)	*Aira praecox *(O)
*Senecio vulgaris *(S)	*Avena fatua *(S)
*Silybium marianum *(S)	*Avena sativa *(S)
*Sonchus oleraceus *(S)	*Bromus hordeaceus *(S)
*Xanthium strumarium *(S)	*Bromus secalinus *(O)
	*Bromus sterilis *(S)
Boraginaceae	*Bromus tectorum *(S)
*Anchusa arvensis *(O)	*Desmazeria rigida *(S)
*Borago officinalis *(O)	*Echinochloa crus-galli *(S)
*Lappula squarrosa *(S)	*Hordeum vulgare *(S)
*Myosotis arvensis *(S)	*Lolium temulentum *(S)
*Myosotis ramosissima *(S)	*Panicum miliaceum *(S)
*Myosotis stricta *(S)	*Phalaris canariensis *(O)
*Plagiobothrys calandrinioides *(S)	*Poa annua *(S)
	*Secale cereale *(O)
Brassicaceae	*Setaria italica *(S)
*Arabidopsis thaliana *(S)	*Setaria verticillata *(S)
*Berteroa incana *(O)	*Setaria virdis *(S)
*Brassica juncea *(O)	*Triticum aestivum *(S)
*Brassica nigra *(O)	*Zea mays *(O)
*Brassica rapa *(O)	
*Cakile edentula *(S)	Polemoniaceae
*Capsella bursa-pastoris *(S)	*Allophyllum gilioides *(S)
*Cardamine hirsute *(S)	*Allophyllum integrifolium *(S)
*Descurainia pinnata *(S)	*Collomia grandiflora *(O)
*Diplotaxis muralis *(O)	*Collomia linearis *(S)
*Erucastrum gallicum *(S)	*Gilia australis *(S)
*Erysimum cheiranthoides *(O)	Gilia capitata *(O)*
*Erysimum repandum (S)*	*Gilia caruifolia *(O)
*Lepidium sativum *(O)	*Gilia clivorum *(S)
*Lepidium campestre *(S)	*Gilia inconspicua *(S)
*Lepidum ruderale *(S)	*Gilia millefoliata *(S)
*Rorippa palustris *(S)	*Gilia sinuata *(S)
*Sinapis alba *(O)	*Gilia tenuiflora *(O)
*Sinapis arvensis *(O)	*Gilia transmontana *(S)
*Sisymbrium officinale *(S)	*Gilia tricolor *(O)
*Thlaspi arvensis *(S)	*Navarretia atrictyloides *(O)
*Thlaspi perfoliatum *(S)	*Navarretia squarrosa *(S)

Caryophyllaceae	Polygonaceae
*Agrostemma githago *(O)	*Fagopyrum esculentum *(O)
*Arenaria serpyllifolia (S)*	*Polygonum aviculare (S)*
*Cerastium nutans (O)*	*Polygonum convolvulus (S)*
*Silene dichotoma (O)*	*Polygonum hydropiper (S)*
*Silene noctiflora (S)*	*Polygonum lapathifolium (S)*
*Spergula arvensis (S)*	*Polygonum persicaria (S)*
*Stellaria media (S)*	
	Ranunculaceae
Fabaceae	*Myosurus minimus (S)*
*Medicago lupulina (O)*	*Ranunculus reptans (O)*
*Trifolium arvense (S)*	*Ranunculus sceleratus *(O)
*Trifolium aureum *(S)	
*Trifolium campestre *(S)	Scrophulariaceae
*Vicia sativa *(S)	*Chaenorrhinum minus *(S)
*Vicia tetrasperma *(O)	*Veronica agrestis *(O)
	*Veronica arvensis *(S)
Lamiaceae	*Veronica peregrina *(S)
*Galeopsis tetrahit *(O)	*Veronica persica *(S)
*Lamium amplesicaule *(S)	
*Lamium purpureum *(S)	Apiaceae
	*Aethusa cynapium *(S)
	*Anethum graveolens *(O)

The contrasts were based on 14 phylogenetically-independent pairs, where each pair consisted of median values of the selfing and outcrossing species within one family, which by definition are species that are more closely related to each other than to any other species in the data set [19]. For plant height, only 13 pairs were used due to missing information. The data were analyzed using a Wilcoxon matched pairs test.

### Greenhouse study

The species included in this study were chosen if there was a known breeding system, if germinable seeds were available, and if a complementary species (i.e. in the same family with the opposite breeding system) was known and could also be obtained as germinable seeds. Seeds were obtained from a variety of sources; Herbiseed, Rancho Santa Ana Botanic Gardens, Chiltern Seeds, S&S Seeds, and the National Plant Germplasm System. Our search lead to an unbiased sample of 25 candidate species, allowing five pair-wise family comparisons and four pair-wise genus comparisons (Table 2).

**Table 2:** List of species, with breeding system (O – outcrosser; S – selfer), used in the greenhouse study .

Family	Family
Species	Species
Asteraceae	Convolvulaceae
*Crepis capillaris *(O)	*Ipomoea hederacea *(S)
*Helianthus annuus *(O)	*Ipomoea purpurea *(O)
*Matricaria maritime *(O)	
*Matricaria matricarioides *(S)	Fabaceae
*Senecio viscosus *(S)	*Lupinus bicolor *(O)
*Senecio vulgaris *(S)	*Lupinus nanus *(S)
	*Lupinus succulentus *(O)
Boraginaceae	*Trifolium hirtum *(S)
*Borago officinalis *(O)	
*Myosotis arvensis *(S)	Lythraceae
	*Cuphea laminuligera *(O)
Brassicaceae	*Cuphea lanceolata *(O)
*Brassica juncea *(O)	*Cuphea lutea *(S)
*Brassica nigra *(O)	
*Capsella bursa-pastoris *(S)	Polemoniaceae
*Cardamine hirsuta *(S)	*Navarretia squarrosa *(S)
*Sinapis alba *(O)	*Phlox drummondii *(O)
*Sinapis arvensis *(O)	

Most species were germinated in 15 cm pots filled to 3 cm below the top with standard potting soil (Promix BX^©^). Pots were placed in a greenhouse and watered daily until the appearance of their first true leaves. Subsequently, they were watered uniformly every second day to ensure that the soil was kept moist. Some species were germinated in a petri-dish in a growth chamber (23°C, 12 hour cycles of light and dark), after which they were transplanted into pots and placed in the greenhouse. Each species was replicated five times, with one plant per pot. Pots were arranged randomly on benches at a density of 1 pot per 0.093 m^2^.

The plants were exposed to 16 hours of daylight each day, with maximal natural light levels at ca 1200 μE. Before sunrise and after sunset, artificial lights (250–300 μE) were used to supplement the light exposure to 16 hours of light per day. The greenhouse was kept at an average temperature of 23.1°C during the day and dropped to 20.0°C at night. The plants were fertilized every 2 weeks with 200 ml per pot of a 2g/L concentration of 20–20–20 N-P-K fertilizer.

For each plant, age at first flower, bud development time, and flower longevity were measured. Emergence of the first pair of true leaves, after the cotyledons, was considered day 1 of the plant's life. Age at first flower was measured in days from day 1 to when the first flower opened on each plant. Bud development time (n = 3 buds per plant) was calculated as the number of days from the first appearance of a new bud until the flower opened. The same three buds on each plant were then monitored every day after opening, and the number of days until the flower senesced (flower longevity) was recorded for each. A flower was considered to be senesced when the corolla wilted, fell apart, or became discolored, as designated by Primack [17]. Any flower that was open for only one day was considered a one-day flower, regardless of whether it was open for the whole day or only part. Flowers in the Asteraceae were considered withered when the whole inflorescence had senesced, rather than the individual florets [17].

For bud development time and flower longevity, the three replicate measurements for each plant were averaged, and then these values for the five replicate plants were averaged to obtain a mean value for the species. The data were analyzed with a standard least squares one-way analysis of variance (ANOVA) model, with a post-hoc contrast between selfers and outcrossers. These analyses were done for each family and genus separately in order to control for phylogeny at these levels. In cases where the data were non-normal, a log-transformation was applied which corrected the distribution.

## Authors' contributions

RS collected the data, performed most of the analyses, participated in the design of the study, and wrote the first draft as a B.Sc.(Hons) thesis. LWA conceived of the study, participated in its design and coordination, and wrote the final draft for submission to BMC. Both authors read and approved the final manuscript.
